# Supplementary material for: Prognostic Factors for Triple-Negative Breast Cancer with Residual Disease after Neoadjuvant Chemotherapy
Source: J Pers Med. 2023 Jan 21;13(2):190. doi: 10.3390/jpm13020190 (PMC9959351; doi:10.3390/jpm13020190)
Supplement: Supplementary file 1 [file jpm-13-00190-s001.zip › jpm-2157288-supplementary.pdf]

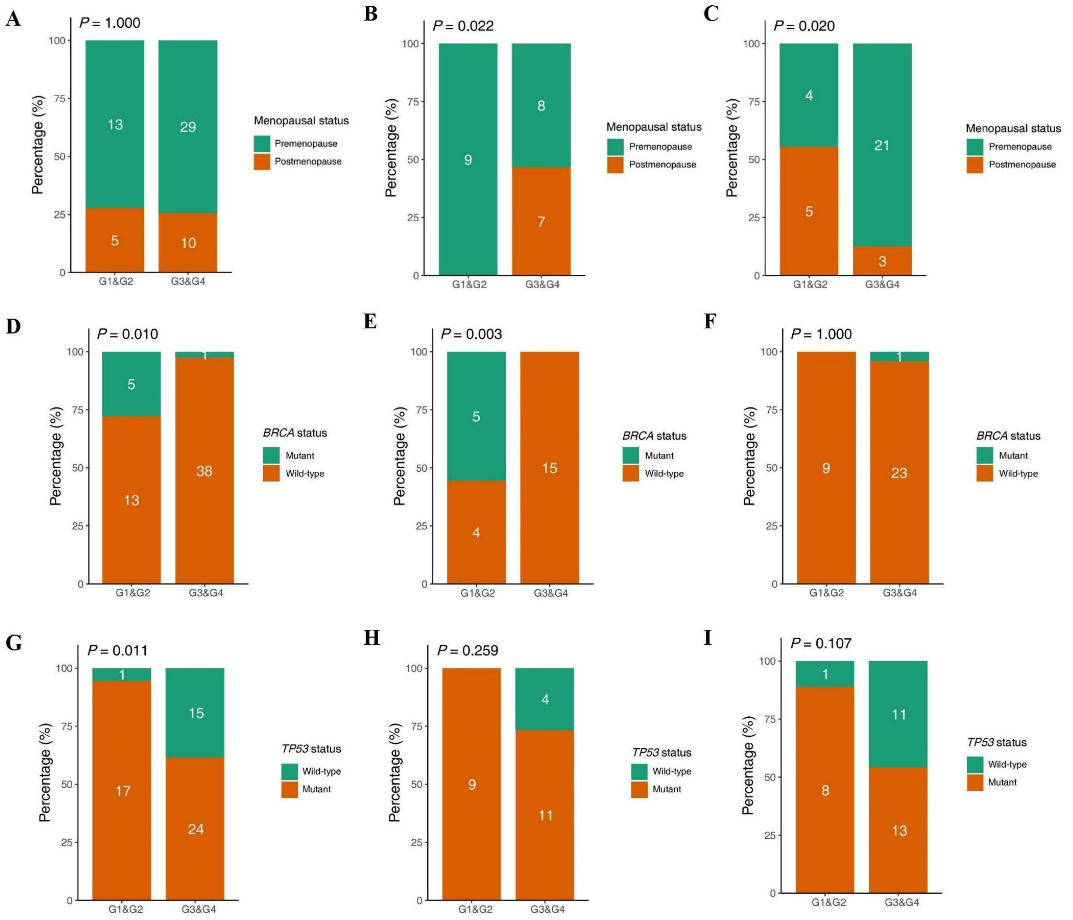

Supplementary Figure S1. Treatment outcomes based on clinical/molecular features. Miller-Payne grades based on menopausal status **A.** in overall patients; **B.** in patients with AP-NAC; **C.** in patients with PP-NAC. Miller-Payne grades based on *BRCA* status **D.** in overall patients; **E.** in patients with AP-NAC; **F.** in patients with PP-NAC. Miller-Payne grades based on *TP53* status **G.** in overall patients; **H.** in patients with AP-NAC; **I.** in patients with PP-NAC. AP anthracycline plus paclitaxel; PP platinum plus paclitaxel; NAC neoadjuvant chemotherapy.

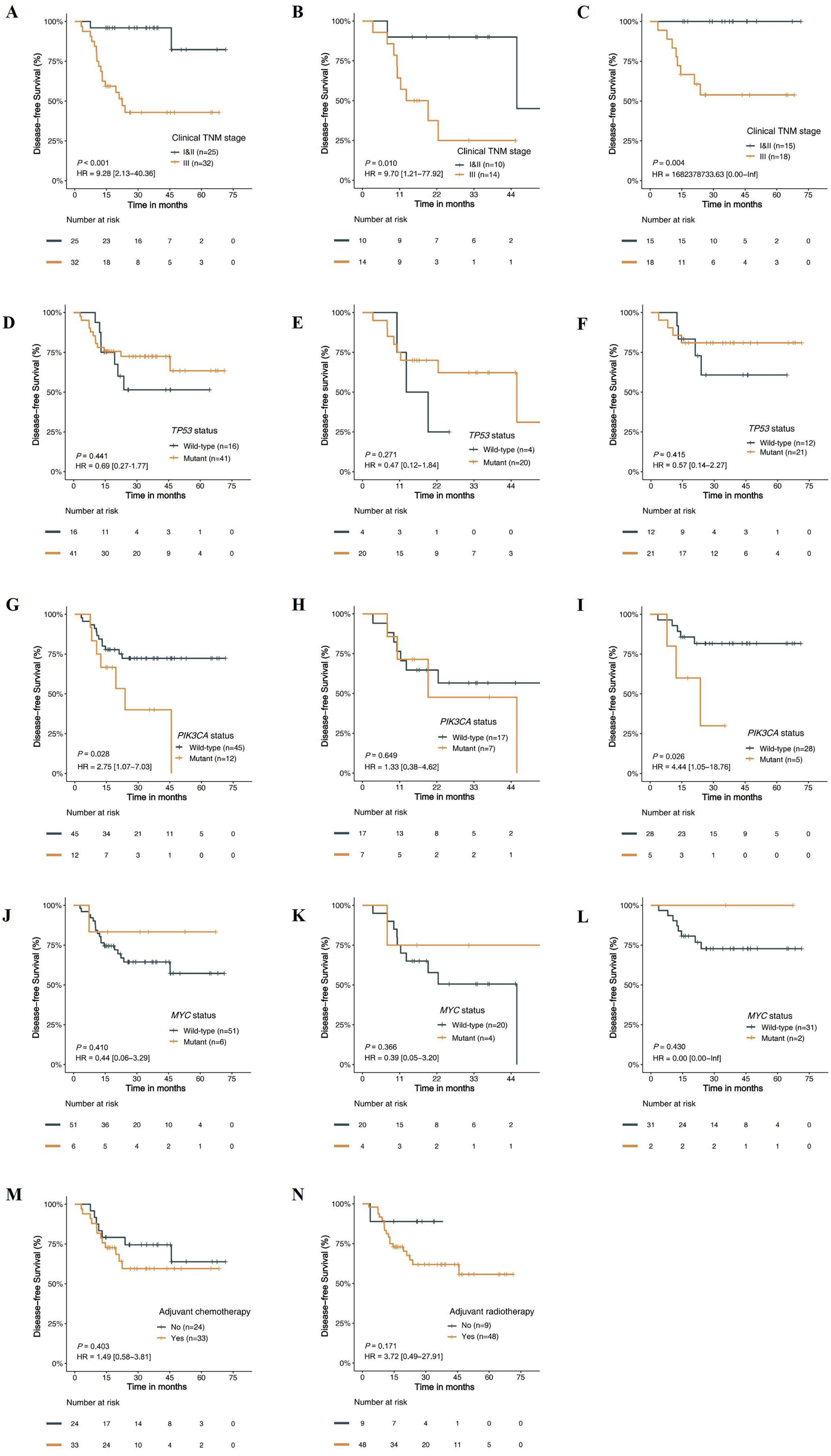
A
Disease−free Survival (%)
Time in months
Clinical TNM stage
I&II (n=25)
III (n=32)
P < 0.001
HR = 9.28 [2.13−40.36]
Number at risk
25 23 16 7 2 0
32 18 8 5 3 0
B
Disease−free Survival (%)
Time in months
Clinical TNM stage
I&II (n=10)
III (n=14)
P = 0.010
HR = 9.70 [1.21−77.92]
Number at risk
10 9 7 6 2
14 9 3 1 1
C
Disease−free Survival (%)
Time in months
Clinical TNM stage
I&II (n=15)
III (n=18)
P = 0.004
HR = 1682378733.63 [0.00−Inf]
Number at risk
15 15 10 5 2 0
18 11 6 4 3 0
D
Disease−free Survival (%)
Time in months
TP53 status
Wild-type (n=16)
Mutant (n=41)
P = 0.441
HR = 0.69 [0.27-1.77]
Number at risk
16 11 4 3 1 0
41 30 20 9 4 0
E
Disease−free survival (%)
Time in months
TP53 status
Wild-type (n=4)
Mutant (n=20)
P = 0.271
HR = 0.47 [0.12−1.84]
Number at risk
4 3 1 0 0
20 15 9 7 3
F
Disease−free Survival (%)
Time in months
TP53 status
Wild-type (n=12)
Mutant (n=21)
P = 0.415
HR = 0.57 [0.14−2.27]
Number at risk
12 9 4 3 1 0
21 17 12 6 4 0
G
Disease−free Survival (%)
Time in months
PIK3CA status
Wild-type (n=45)
Mutant (n=12)
P = 0.028
HR = 2.75 [1.07−7.03]
Number at risk
45 34 21 11 5 0
12 7 3 1 0 0
H
Disease−free Survival (%)
Time in months
PIK3CA status
Wild-type (n=17)
Mutant (n=7)
P = 0.649
HR = 1.33 [0.38−4.62]
Number at risk
17 13 8 5 2
7 5 2 2 1
I
Disease−free Survival (%)
Time in months
PIK3CA status
Wild-type (n=28)
Mutant (n=5)
P = 0.026
HR = 4.44 [1.05−18.76]
Number at risk
28 23 15 9 5 0
5 3 1 0 0 0
J
Disease−free Survival (%)
Time in months
MYC status
Wild-type (n=51)
Mutant (n=6)
P = 0.410
HR = 0.44 [0.06−3.29]
Number at risk
51 36 20 10 4 0
6 5 4 2 1 0
K
Disease−free Survival (%)
Time in months
MYC status
Wild-type (n=20)
Mutant (n=4)
P = 0.366
HR = 0.39 [0.05−3.20]
Number at risk
20 15 8 6 2
4 3 2 1 1
L
Disease−free Survival (%)
Time in months
MYC status
Wild-type (n=31)
Mutant (n=2)
P = 0.430
HR = 0.00 [0.00−Inf]
Number at risk
31 24 14 8 4 0
2 2 2 1 1 0
M
Disease−free Survival (%)
Time in months
Adjuvant chemotherapy
No (n=24)
Yes (n=33)
P = 0.403
HR = 1.49 [0.58−3.81]
Number at risk
24 17 14 8 3 0
33 24 10 4 2 0
N
Disease−free Survival (%)
Time in months
Adjuvant radiotherapy
No (n=9)
Yes (n=48)
P = 0.171
HR = 3.72 [0.49−27.91]
Number at risk
9 7 4 1 0 0
48 34 20 11 5 0

Supplementary Figure S2. Survival analysis according to different clinicopathologic and molecular features. DFS based on clinical TNM stage **A.** in overall patients; **B.** in patients with AP-NAC; **C.** in patients with PP-NAC. DFS based on *TP53* status **D.** in overall patients; **E.** in patients with AP-NAC; **F.** in patients with PP-NAC. DFS based on *PIK3CA* status **G.** in overall patients; **H.** in patients with AP-NAC; **I.** in patients with PP-NAC. DFS based on *MYC* status **J.** in overall patients; **K.** in patients with AP-NAC; **L.** in patients with PP-NAC. DFS based on **M.** adjuvant chemotherapy; **N.** adjuvant radiotherapy. DFS disease-free survival; AP anthracycline plus paclitaxel; PP platinum plus paclitaxel; NAC neoadjuvant chemotherapy.
